# A method for soil moisture probes calibration and validation of satellite estimates

**DOI:** 10.1016/j.mex.2017.07.004

**Published:** 2017-07-18

**Authors:** Mauro Holzman, Raúl Rivas, Facundo Carmona, Raquel Niclòs

**Affiliations:** a Instituto de Hidrología de Llanuras “Dr. Eduardo J. Usunoff” (UNCPBA-CIC-MA), CONICET, UNCPBA-IHLLA, Azul B7300, Argentina; b Instituto de Hidrología de Llanuras “Dr. Eduardo J. Usunoff” (UNCPBA-CIC-MA), Comisión de Investigaciones Científicas de la provincia de Buenos Aires, Tandil B7000, Argentina; c Instituto de Hidrología de Llanuras “Dr. Eduardo J. Usunoff” (UNCPBA-CIC-MA), CONICET, UNCPBA-IHLLA, Tandil B7000, Argentina; d Department of Earth Physics and Thermodynamics, University of Valencia, 46100 Burjassot, Spain

**Keywords:** Soil water content, Soil Moisture and Ocean Salinity (SMOS), Land surface processes, Remote sensing

## Abstract

Optimization of field techniques is crucial to ensure high quality soil moisture data. The aim of the work is to present a sampling method for undisturbed soil and soil water content to calibrated soil moisture probes, in a context of the SMOS (Soil Moisture and Ocean Salinity) mission MIRAS Level 2 soil moisture product validation in Pampean Region of Argentina. The method avoids soil alteration and is recommended to calibrated probes based on soil type under a freely drying process at ambient temperature. A detailed explanation of field and laboratory procedures to obtain reference soil moisture is shown. The calibration results reflected accurate operation for the Delta-T thetaProbe ML2x probes in most of analyzed cases (RMSE and bias ≤ 0.05 m^3^/m^3^). Post-calibration results indicated that the accuracy improves significantly applying the adjustments of the calibration based on soil types (RMSE ≤ 0.022 m^3^/m^3^, bias ≤ −0.010 m^3^/m^3^).

• A sampling method that provides high quality data of soil water content for calibration of probes is described.
• Importance of calibration based on soil types.
• A calibration process for similar soil types could be suitable in practical terms, depending on the required accuracy level.

## Graphical abstract

**Figure:**
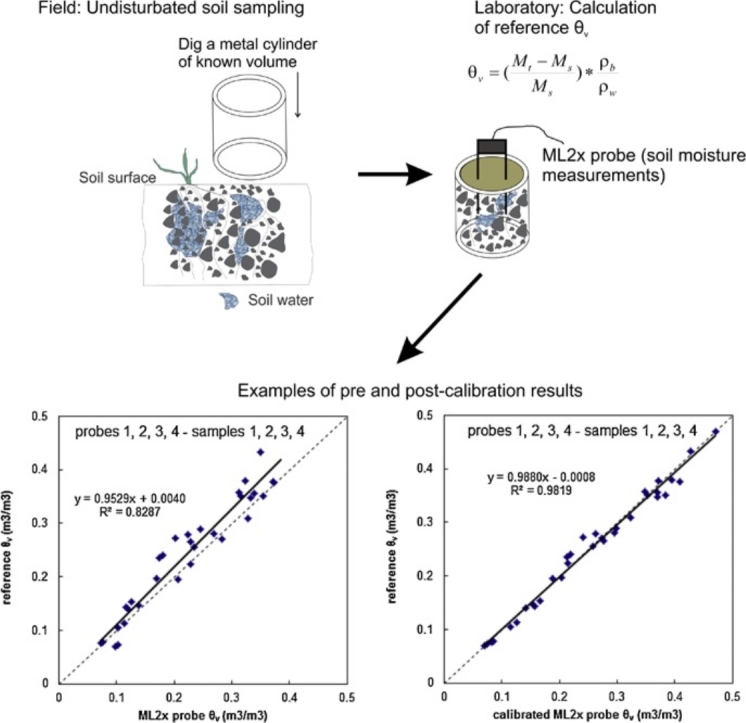

## Method details

The United Nations recognize the critical role of soils in sustainable development, given that soils contribute to ecosystem services related to several of the United Stations Sustainable Development Goals (e.g. food security in developing countries, health, water security/resources, biodiversity) [1]. Soil moisture is an essential component of the soil-vegetation-atmosphere system determining physical processes (e.g. water cycle and energy balance, land-atmosphere interactions) and the functioning of plants and other soil biota [2]. The interdisciplinary study of soil moisture is crucial to understand links between soils and climate and to improve climate models and agricultural production, given the impact on crop yield and food security [2], [3]. It can show a high spatial variability due to diverse factors like topography, ground water level, soil type or vegetation cover [4], [5] and these variations produce significant changes in regional runoff, crop productivity or groundwater recharge, among others. In [3], [4] we showed the spatial impact of soil water deficit and excess on the main crops of Pampean Region of Argentina, one of the major grain producers of the world.

On the other hand, in the last decades the pressure on water resources managers has been increasing to maintain soil water and to maximize the productivity of natural and agricultural systems. In this sense, diverse satellite missions have been designed to monitor spatially surface soil moisture (e.g. Soil Moisture and Ocean Salinity-SMOS, Soil Moisture Active Passive −SMAP). Besides, significant efforts have been done to validate the retrieved soil moisture data, including expensive field campaigns. In this context, measured data are assumed to represent the truth and are used for adjusting models and decisions [1]. However, efforts should be done to optimize field techniques to ensure high quality data. In [6] a campaign for a SMOS soil moisture product validation in Pampean Region of Argentina was carried out. The field/laboratory techniques were briefly described and the more detailed process included in this work should be useful for different studies to understand soil-vegetation-atmosphere processes. These aspects should be determining in a context of climate change and the growing world population and its food needs.

The aim of the work is to present a sampling method for undisturbed soil and soil water content to calibrate soil moisture probes (in this case, Delta-T thetaProbe ML2x probes) and validation of satellite estimates. The calibration process for specific soil types was carried out. The method is highly recommended to calibrated soil moisture probes based on soil type under a freely drying process at ambient temperature.

## Materials and methods

### Field sampling procedure

The Pampean Region covers the most productive area of Argentina whose dominant soil order is Mollisol, characterized by a fertile mollic epipedon (see www.soils.org/publications/soils-glossary#) [7]. Also, Argiudoll is the main soil great group of the humid and sub-humid area of the region, and the organic matter content of the A horizon varies approximately between 2% (Córdoba province) and 5% (South of Buenos Aires province) [8]. The campaign was designed to cover representative soils at regional scale, covering Córdoba and Buenos Aires provinces. During the campaign (February 2013), handheld soil moisture measurements were carried out with ML2 x probes along three simultaneous parallel transects at the SMOS overpass time for each study parcel. In each transect, one team measured every 10 m for a period of around 15 min centered at the SMOS overpass, first moving towards the center of the parcel in parallel tracks and then two people branched out at 90° in opposite directions until the parcel limit. Approximately 30 measurements were collected in each transect. The Delta-T thetaProbe ML2 x measures volumetric soil moisture content (θ_v_), by the method of responding to changes in the apparent dielectric constant, which is proportional to soil moisture content. The accuracy level specified by manufacturer are: ±0.05 m^3^/m^3^ (0 to 70 °C) using the supplied soil calibration; ±0.01 m^3^/m^3^ (0 to 40 °C) and ±0.02 m^3^/m^3^ (40 to 70 °C) after calibration to a specific soil type [9].

**Fig. 1:**
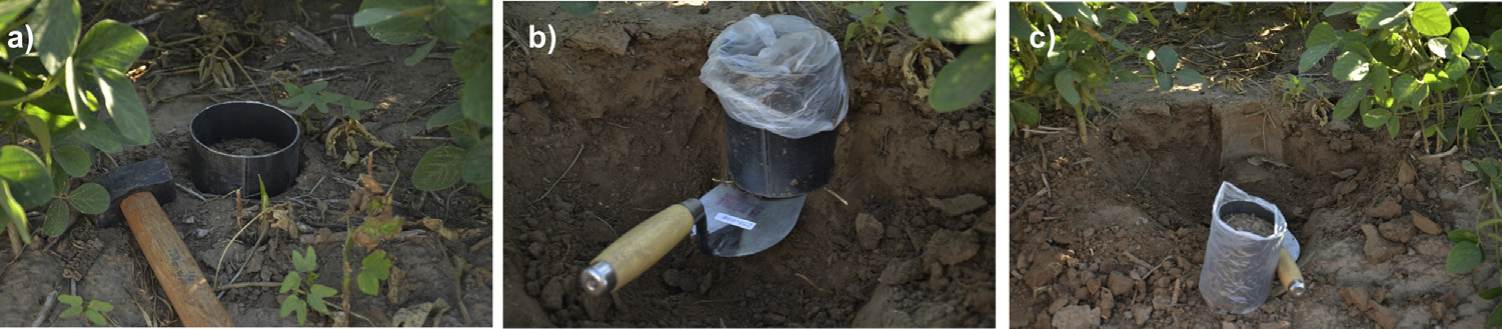
Sampling of undisturbed soil (10 cm depth) using a metal cylinder.

The validation of the SMOS MIRAS Level 2 soil moisture product (SMOS MIR_SMUDP2, v5.51) was realized using these data. In each parcel, a representative and non-altered soil sample (10 cm depth) was collected digging a metal cylinder of known volume (Fig. 1a). In this case, the dimensions of the cylinders were: radius = 4.9 cm, height ≈ 10.7 cm, volume≈ 800 cm^3^. Then, the sample is cut at the base of the cylinder and both extremes of the tube are covered avoiding sample loss (Fig. 1b, c). Crumbling and drift of soil can be more frequent in poorly structured or sandy soils [10]. It should be noted that most of soil sampling methods implies soil modification (e.g. at collecting time or when the probe is inserted several times). However, it changes soil water retention capacity (e.g. soil structure). Undisturbed samples are required if a high level of accuracy is needed.

Table 1 shows four different soil types sampled under rainfed soybean crop to calibrate the soil moisture probes and evaluate their uncertainty. Parcels corresponding to samples 1 and 2 were located in Córdoba province and samples 3 and 4, in Buenos Aires province (county: Tandil).

**Table 1:** Soil type, textural classes and organic matter for each analyzed sample.

Sample ID	Lat/Long	Parcel soil type	Parcel soil textural class	(Clay, Silt, Sand) (%)	Organic matter (%)
1	32°29′26′’S; 62°43′37′’W	Typic Haplustoll	Loam	(22,46,32)	2.2
2	32°58′56′’S; 62°28′42′’W	Typic Argiudoll	Clay Loam	(29,45,26)	3.0
3	37°18′32′’S; 58°58′40′’ W	Typic Argiudoll	Loam	(24,39,37)	5.4
4	37°20′44′’S; 58°34′4.3′’W	Typic Argiudoll	Silty loam	(25,51,24)	4.8

### Laboratory procedure

Reference soil moisture content (*θ_ν_*) in soil samples was obtained using the gravimetric method, because of its low cost and high accuracy. In the laboratory, the base of each cylinder was closed with a paper filter (could be a metal filter) that allows only the descendent movement of water. Then the samples were saturated and, since that moment, they were freely dried at ambient temperature (approximately 20 °C) by evaporation process. The weight of cylinder + soil + probe was measured once a day during the drying process. Simultaneously, *θ_ν_* was measured using the ML2x probes. This process was continued up to the lowest sample weight (constant during three days). It should be noted that each ML2x was fixed inside the soil throughout the process to avoid sample loss. After that, the bulk density (*ρ_b_*) was measured for each of three samples as the ratio between dry mass (*M_s_*) and volume of the sample (*V_t_*):

(1) $$\rho_{b}=\frac{M_{s}}{V_{t}}$$

The dry mass was obtained at the end of the drying process following the typical gravimetric method drying the soil samples in an oven at 105 °C for 48 h [11], subtracting the weight of cylinder + probe. The volume of soil was calculated according to the dimensions of the cylinders.

Knowing the bulk density, reference *θ_ν_* was obtained:

(2) $$\theta_{v}=(\frac{M_{t}-M_{s}}{M_{s}})*\frac{\rho_{b}}{\rho_{w}}$$

where *M_t_* is the mass of soil plus water and *ρ_w_* is the water density.

Finally, reference *θ_ν_* and *θ_ν_* measured with ML2x were compared and regression equations were calculated for each soil sample. After calibration, the corresponding validation errors (RMSE and bias) were obtained using a different data set. Negative/positive bias values indicate overestimation/underestimation, respectively. Each of the three ML2x probes was calibrated following the same process.

### Method validation

**Fig. 2:**
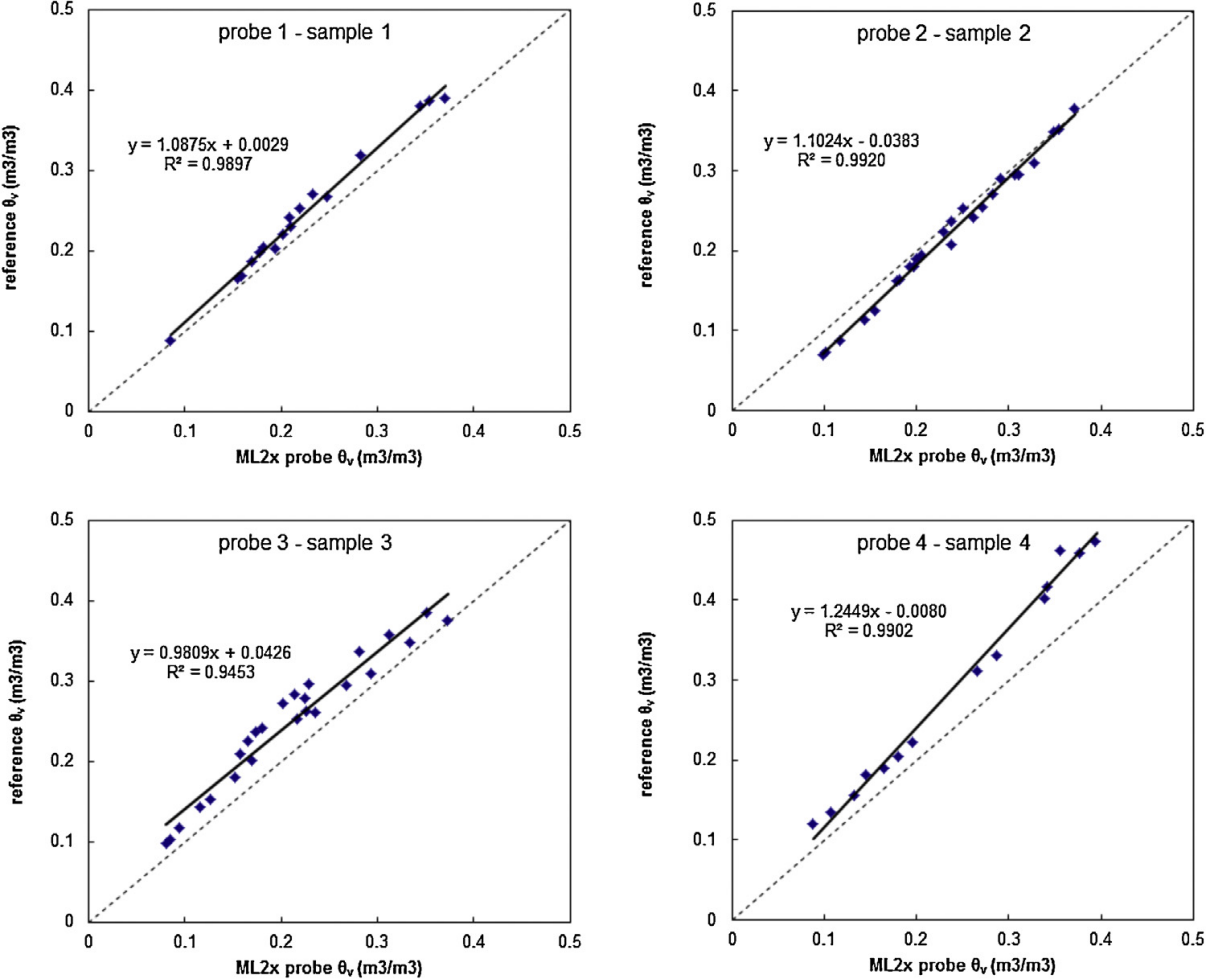
Results of laboratory calibration procedure carried out for the four ML2x probes and four different soil types. Reference *θ_ν_* was obtained by gravimetric method.

In order to validate the presented method, linear regression equations between reference *θ_ν_* and measured *θ_ν_* were obtained, which showed high correlation between data (Fig. 2). Fig. 2 includes the four described samples, but other samples showed similar results (please see also [6]). The lowest coefficient of determination value was observed in sample 3. Also, the validation results showed the highest RMSE and bias in sample 4 (Table 2). These samples had high organic matter content and was taken in a different region in comparison with samples 1 and 2 (Table 1). This could reflect the importance of the organic matter to define soil type in relation to processes of soil water. Also, the high organic matter content could be a source of uncertainty for the measured soil moisture. Taking into account that the poorest results were obtained in sample 3 and 4, the calibration process considering similar soils should be useful in practical terms. On the other hand, it should be noted that the adjustments are different for each probe and soil and reflect that a calibration for each probe should be needed depending on the required accuracy level (e.g. irrigation purposes at field scale, soil water repellency and erosion process).

**Table 2:** Pre and post-calibration errors for the four ML2x. Numbers in brackets indicate post-calibration error values.

	Probe 1–sample 1	Probe 2 − sample 2	Probe 3 − sample 3	Probe 4 − sample 4
n	8	8	9	8
RMSE	0.011 (0.010)	0.017 (0.007)	0.046 (0.022)	0.054 (0.014)
Bias	0.008 (−0.009)	−0.013 (0)	0.040 (0.002)	0.048 (−0.008)

The validation results reflected accurate operation for the four ML2x probes in most cases, with Root Mean Square Error (RMSE) values lower than 0.046 m^3^/m^3^ (except in sample 4, RMSE = 0.054 m^3^/m^3^), which is in accordance with the manufacturer specifications using the supplied soil calibration (0.05 m^3^/m^3^) (Table 2) [9]. Probes 1 and 2 showed a very good performance, with accuracy level similar to the specified by manufacturer for calibrated probes. The bias values suggested underestimation of the soil water content. It should be noted that post-calibration RMSE and bias values indicated considerable higher accuracy applying the adjustments of the calibration process.

**Fig. 3:**
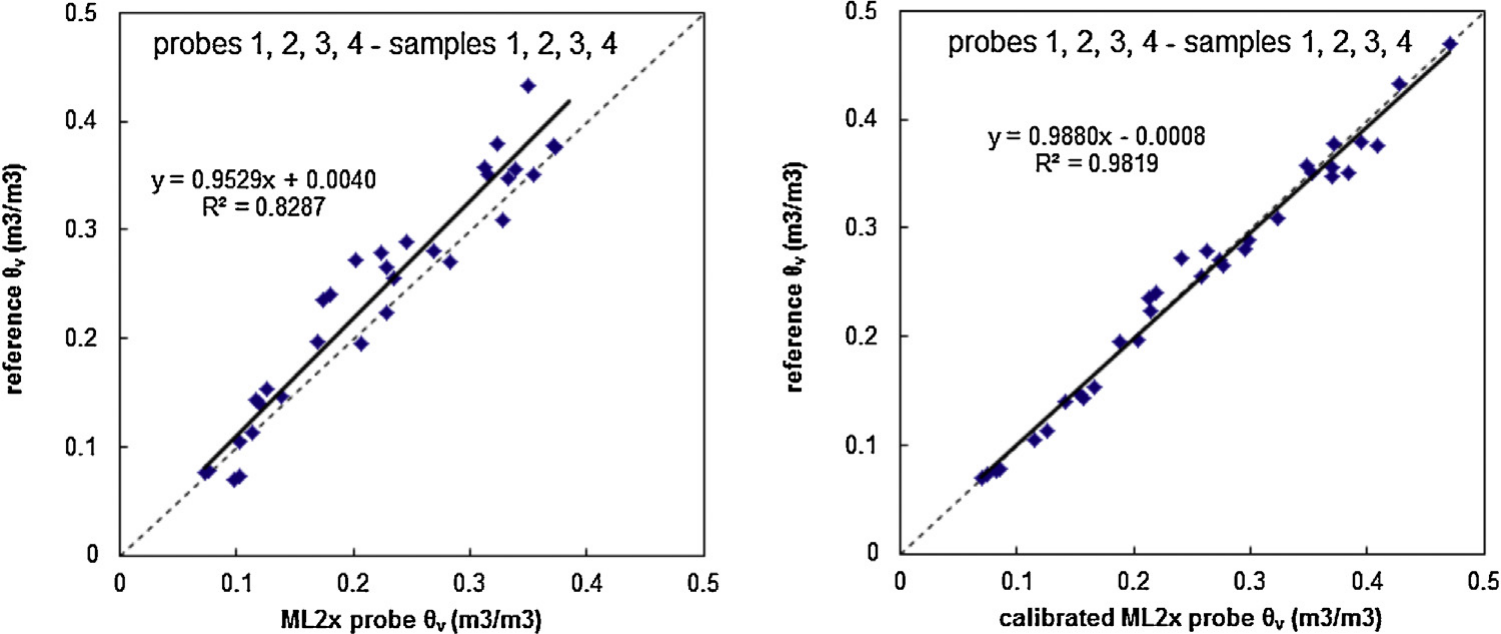
Comparison between reference *θ_ν_* and measured *θ_ν_* using the supplied calibration by manufacturer (left) and calibrated probes according to soil type (right).

The comparison between reference *θ_ν_* and measured *θ_ν_* using the supplied calibration by manufacturer and calibrated probes, considering the data set of validation (n = 33), shows the importance of calibration (Fig. 3). Higher R^2^ value was obtained with probes calibrated according to soil type.

## Concluding remarks

The proposed sampling method to measure soil water content in undisturbed soil and to calibrate soil moisture probes provides high quality data. One advantage of the method is that it needs no trained personnel and typical laboratory materials. The work shows its suitability in a context of a field campaign to evaluate the SMOS MIRAS Level 2 soil moisture product (SMOS MIR_SMUDP2, v5.51) in an area of rainfed crops of Argentina using Delta-T thetaProbe ML2x soil moisture probes.

Results of calibration process showed that, in most cases, the probes meet the level of accuracy specified by the manufacturer using the supplied soil calibration. Such level of accuracy is suitable for satellite validation purpose at regional scale (e.g. SMOS: 40 km spatial resolution, whose mission’s accuracy goal is ±0.04 m^3^/m^3^). After the calibration process based on soil types, that accuracy improves significantly. Previous works have reported that common issues of satellite products validation are the unknown errors and inconsistencies of in-situ measurements induced by erroneous calibration. This work should contribute to enhancing not only the soil moisture assessments of current and future remote sensing missions, but also networks of in-situ monitoring stations (e.g. see www.conae.gov.ar/index.php/english/satellite-missions/saocom/introduction). Also, this work expects to give a general contribution to soil science as studies of spatial fluctuations of soil moisture to mitigate economic and social impacts of extreme events (e.g. droughts or intense precipitation, soil erosion).

It should be noted the importance of calibration process to obtain reliable data of soil moisture for irrigation or water management purposes at field scale. In fact, an inter-calibration could be needed if several probes are simultaneously used. Also high organic matter content could be a source of uncertainty for the measured soil moisture. On the other hand, depending on the objective of the work, results could suggest that a calibration process for similar soil types at regional scale should be suitable in practical terms, saving time/cost in comparison with a calibration for each soil.
